# Supplementary material for: Sitagliptin Induces Tolerogenic Human Dendritic Cells
Source: Int J Mol Sci. 2023 Nov 27;24(23):16829. doi: 10.3390/ijms242316829 (PMC10706581; doi:10.3390/ijms242316829)
Supplement: Supplementary file 1 [file ijms-24-16829-s001.zip › ijms-2624451-supplementary.pdf]

Supplementary Tables and Figures

Supplementary Table S1. Dose-dependent effects of sitagliptin on selected parameters of the phenotype and functions of MoDCs (preliminary data)

Parameters	Sitagliptin (sita 0d protocol) (μg/mL)			
	0	100	250	500
CD14 (%)	5.4 ± 1.1	6.2 ± 1.2	30.1 ± 4.8**	34.2 ± 3.3**
CD83 (%)	64.2 ± 5.0	60.1 ± 6.2	28.3 ± 4.0**	35.2 ± 3.8**
CD86 (MFI)	24126 ± 2300	19499 ± 3342*	12160 ± 2400**	5143 ± 675***
ILT4 (%)	26.1 ± 3.1	28.2 ± 3.6	39.6 ± 6.4*	70.4 ± 5.8***
IDO1 (%)	37.8 ± 4.0	47.1 ± 3.6*	46.6 ± 4.8*	65.6 ± 5.5**
CD26 (%)	26.2 ± 3.0	NT	53.3 ± 4.2**	49.2 ± 2.8**
IL-12p70 (pg/mL)	2122 ± 220	1726 ± 152*	1890 ± 206	1245 ± 148**
IL-23 (pg/mL)	1976 ± 208	1864 ± 214	1564 ± 126*	886 ± 125**
IL-27 (pg/mL)	2806 ± 293	2950 ± 322	2726 ± 322	2140 ± 205*
IL-1β (pg/mL)	32.2 ± 4.0	36.6 ± 3.9	29.1 ± 4.0	18.4 ± 2.2**
IL-10 (pg/mL)	712 ± 84	684 ± 72	956 ± 88*	802 ± 99
TGF-β (pg/mL)	204 ± 31	288 ± 35**	272 ± 30**	310 ± 36**
Proliferation (%)	56.2 ± 4.6	48.1 ± 3.8*	41.4 ± 5.0**	32.6 ± 4.0 **

The experiment was performed with mature MoDCs, except for the expression of CD14 on immature MoDCs. Values are presented as mean ± SD (triplicates) of one experiment. * $p < 0.05$; ** $p < 0.01$; *** $p < 0.005$ compared to control cultures without sitagliptin. NT – Non-tested; The results obtained with 500 μg/mL of sitagliptin were not included in the final experiments.

Supplementary Table S2. Viability of MoDCs in control and sitagliptin-treated cultures

imMoDCs		Trypan blue + cells (%)	Total viability (%)
	Control	4.1 ± 2.2	81.6 ± 4.2
	Sita 0d protocol	5.2 ± 1.7	78.4 ± 4.5
	Sita 4d protocol	4.8 ± 1.3	82.6 ± 3.9
mMoDCs			
	Control	5.9 ± 1.6	72.4 ± 4.8
	Sita 0d protocol	5.5 ± 1.9	70.2 ± 5.1
	Sita 4d protocol	6.4 ± 1.7	69.2 ± 5.0

The viability of MoDCs in a 5-day culture period was assessed by staining the cells with Trypan blue. Dead (Trypan blue+ cells) were presented as mean % ± SD (n = 3 different cultures). Total viability (% of survival cells in culture relative to the initial number of cells used as 100%) was determined as follows: number of cells in culture – number of dead cells/ number of initial cells x 100. The differences between sitagliptin-treated MoDCs and corresponding control MoDCs for both parameters were not statistically significant ($p > 0.05$).

Supplementary Table S3. The total number of T cells recovered at the end of the co-culture period

Co-culture		N^0 of T cells (Th polarization protocol) (x 10^5)	N^0 of T cells (Tregs protocol) (x 10^5)
imMoDC/T cells			
	Control	1.64 ± 0.14	1.34 ± 0.22
	Sita 0d	1.22 ± 0.22*	1.24 ± 0.34
	Sita 4d	1.46 ± 0.26	1.18 ± 0.26
mMoDC/T cells			
	Control	2.24 ± 0.18	2.48 ± 0.33
	Sita 0d	1.65 ± 0.23*	2.08 ± 0.28
	Sita 4d	1.77 ± 0.24*	2.24 ± 0.25

The total number of T cells in MoDC/T-cell co-cultures was calculated manually after 4 days (Th polarization protocol) or 6 days (Tregs protocol). Values are given as mean ± SD of triplicates of one representative experiment. * $p < 0.05$ compared to corresponding controls.

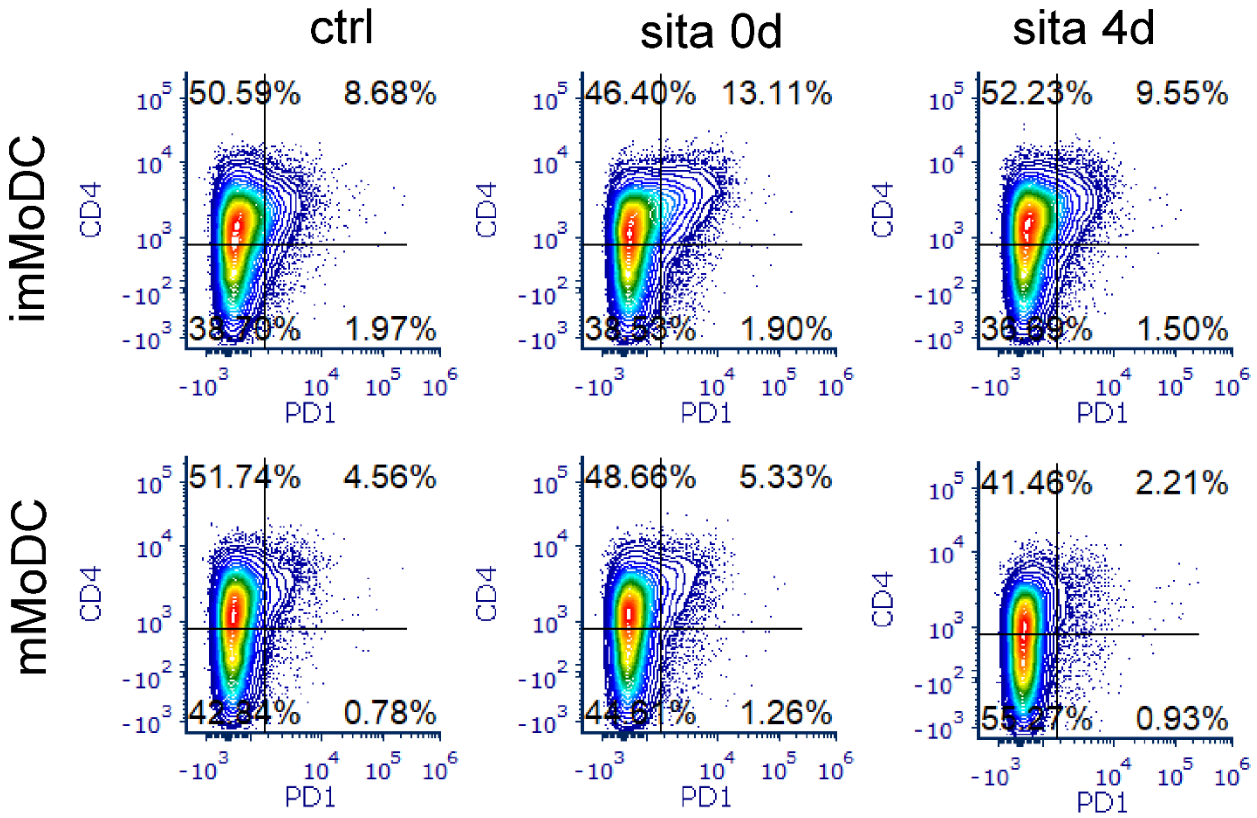

Supplementary Figure S1. The effect of MoDCs treated with sitagliptin (500 µg/mL) on the expression of PD1 on T cells in co-culture. The plots, from one experiment out of three ones with similar results, show the percentages of CD4+PD1+ T cells.

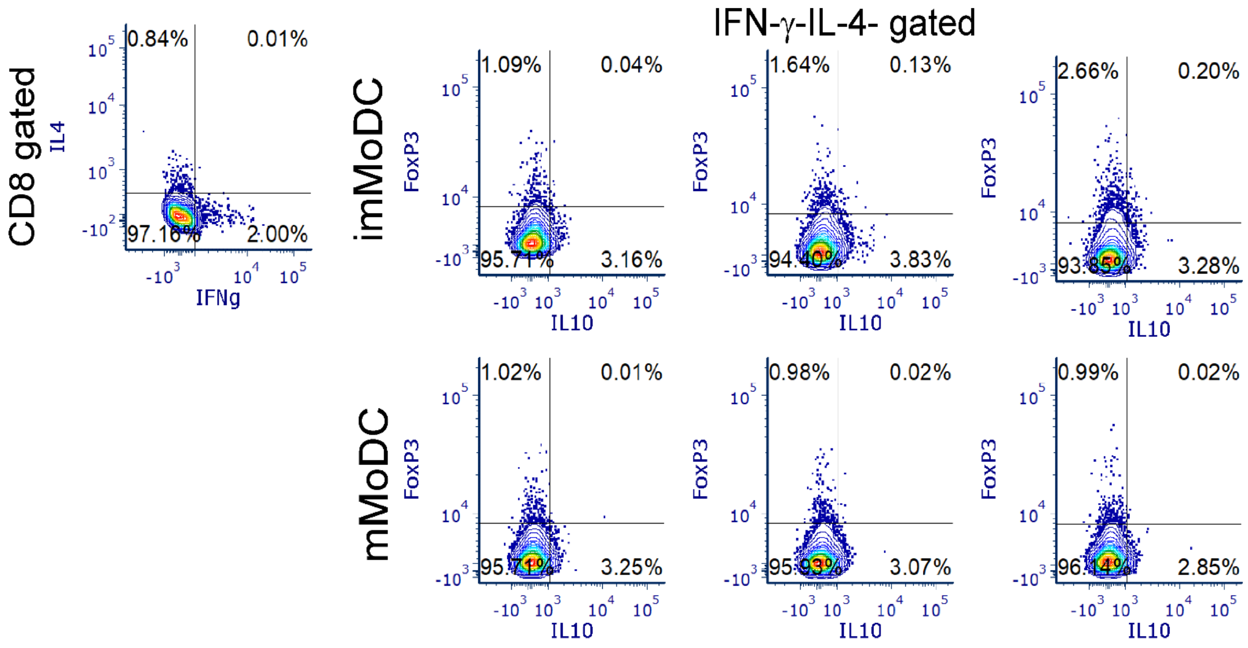

Supplementary Figure S2. The effect of MoDCs treated with sitagliptin (500 μg/mL) on the induction of regulatory cells within the CD8+ T-cell population. The cells were identified as CD8+Foxp3-IL-10+ T cells within the IFN-γ-IL-4-CD8+ T-cell population. The results (flow cytometric plots) are from one representative experiment, out of three ones with similar findings.

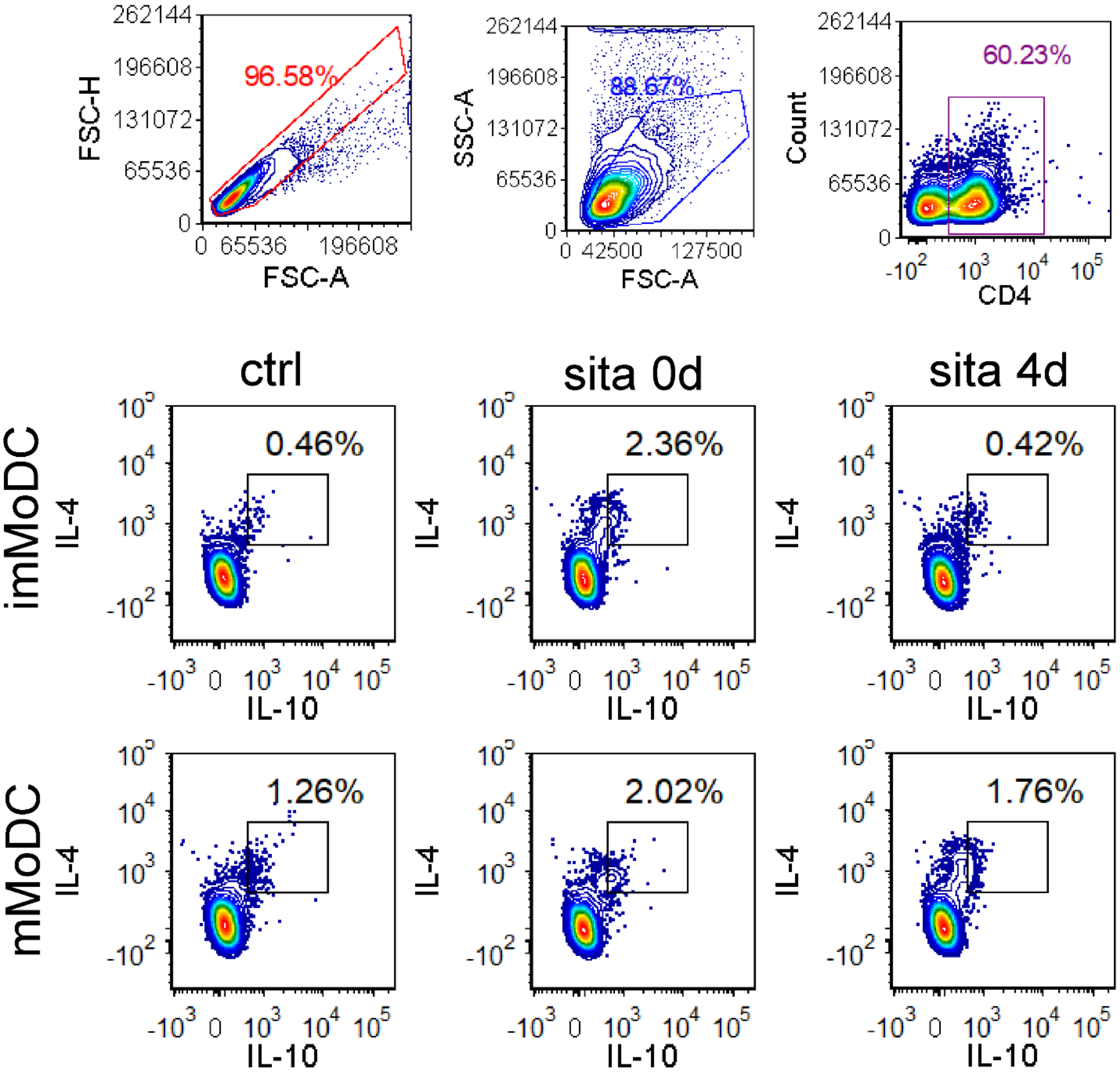

Supplementary Figure S3. The effect of MoDCs treated with sitagliptin (500 μg/mL) on the expression of IL-10 in the Th2 subset. The plots, from one experiment out of three ones with similar results, show the percentages of IL-4+IL-10+ T cells.

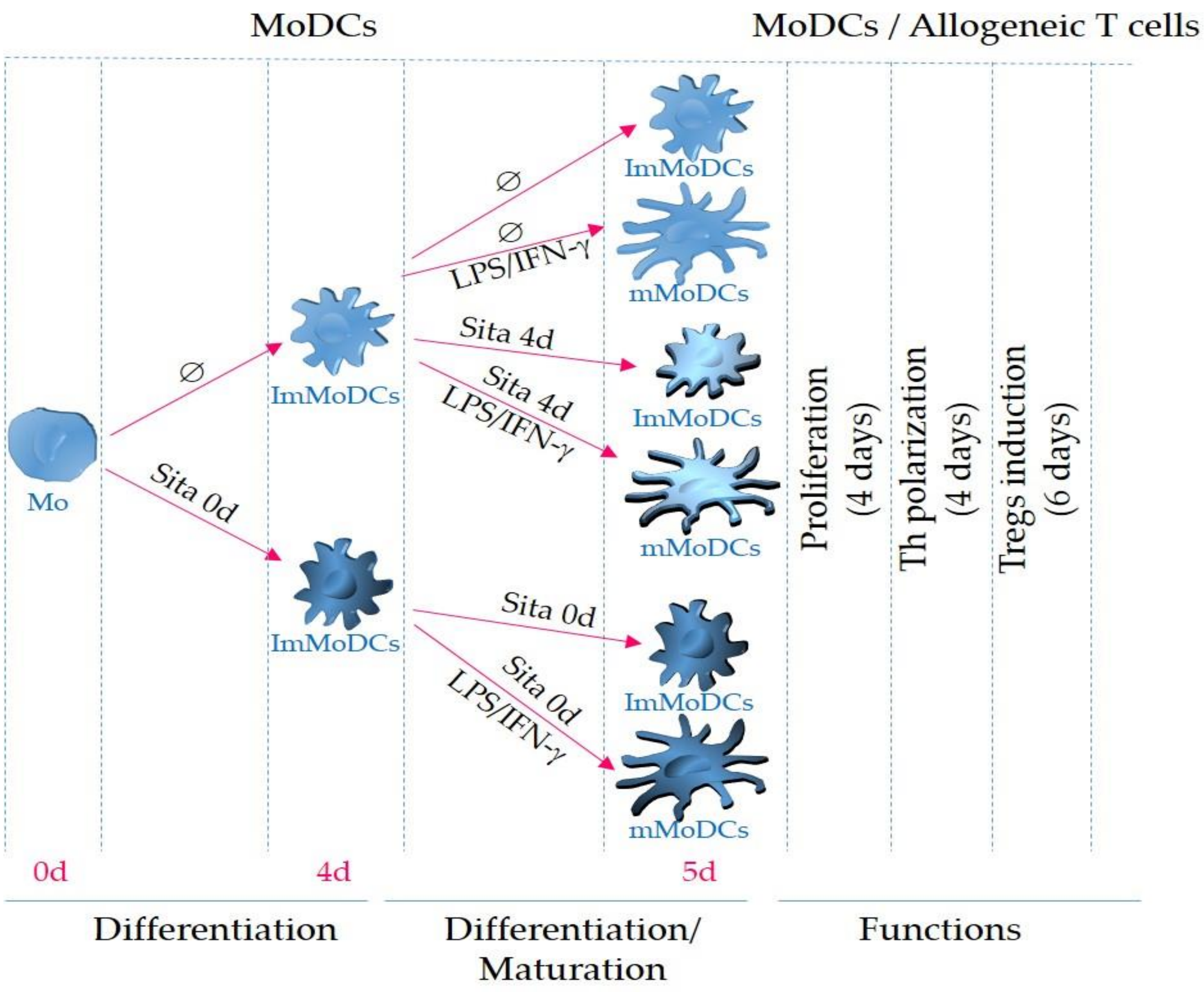

Supplementary Figure S4. Schematic presentation of the methodology for the generation of MoDCs and testing their functions.
